# Chondroitin Sulfate as a Potential Modulator of the Stem Cell Niche in Cornea

**DOI:** 10.3389/fcell.2020.567358

**Published:** 2021-01-12

**Authors:** Sean Ashworth, Jodie Harrington, Greg M. Hammond, Kiranjit K. Bains, Elena Koudouna, Anthony J. Hayes, James R. Ralphs, Justyn W. Regini, Robert D. Young, Ryuhei Hayashi, Kohji Nishida, Clare E. Hughes, Andrew J. Quantock

**Affiliations:** ^1^Structural Biophysics Group, School of Optometry and Vision Sciences, College of Biomedical and Life Sciences, Cardiff University, Cardiff, United Kingdom; ^2^School of Biosciences, College of Biomedical and Life Sciences, Cardiff University, Cardiff, United Kingdom; ^3^Department of Stem Cells and Applied Medicine, Osaka University Graduate School of Medicine, Osaka, Japan; ^4^Department of Ophthalmology, Osaka University Graduate School of Medicine, Osaka, Japan

**Keywords:** cornea, chondroitin sulfate, proteoglycan, glycosaminoglcyan, stem cell niche, human iPS cells

## Abstract

Chondroitin sulfate (CS) is an important component of the extracellular matrix in multiple biological tissues. In cornea, the CS glycosaminoglycan (GAG) exists in hybrid form, whereby some of the repeating disaccharides are dermatan sulfate (DS). These CS/DS GAGs in cornea, through their presence on the proteoglycans, decorin and biglycan, help control collagen fibrillogenesis and organization. CS also acts as a regulatory ligand for a spectrum of signaling molecules, including morphogens, cytokines, chemokines, and enzymes during corneal growth and development. There is a growing body of evidence that precise expression of CS or CS/DS with specific sulfation motifs helps define the local extracellular compartment that contributes to maintenance of the stem cell phenotype. Indeed, recent evidence shows that CS sulfation motifs recognized by antibodies 4C3, 7D4, and 3B3 identify stem cell populations and their niches, along with activated progenitor cells and transitional areas of tissue development in the fetal human elbow. Various sulfation motifs identified by some CS antibodies are also specifically located in the limbal region at the edge of the mature cornea, which is widely accepted to represent the corneal epithelial stem cell niche. Emerging data also implicate developmental changes in the distribution of CS during corneal morphogenesis. This article will reflect upon the potential roles of CS and CS/DS in maintenance of the stem cell niche in cornea, and will contemplate the possible involvement of CS in the generation of eye-like tissues from human iPS (induced pluripotent stem) cells.

## Introduction

Proteoglycans form key components of the extracellular matrix, typically consisting of a protein core with one or more covalently attached glycosaminoglycan (GAG) chains. These molecules play vital roles in cell-cell signaling, tissue homeostasis and wound healing. Chondroitin sulfate/dermatan sulfate (CS/DS) and certain sulfation motifs of these GAG species are present in the stem cell niche in various tissues (Hayes et al., 2008, 2016; Caterson, 2012; Melrose et al., 2012), and reportedly influence progenitor and stem cell function in composite tissue scaffolds (Farrugia et al., 2018). The importance of CS/DS as a structural extracellular matrix component in the cornea is fairly well-established (Lewis et al., 2010; Chen and Birk, 2011; Parfitt et al., 2011; Chen et al., 2014, 2015), but its potential role in the maintenance and development of the stem cell niche in cornea has been little studied until recently.

The cornea is the transparent tissue at the front of the eye. In humans, it is ~0.5 mm thick and 11–12 mm in diameter, wherein it merges with the white sclera of the eye at an anatomical region known as the limbus. The bulk of the cornea is composed of a collagen-rich extracellular matrix – the corneal stroma – that contains ~250 stacked and interwoven sheets or lamellae, made up of uniformly thin (~30 nm diameter), and regularly-spaced, hybrid type I/V collagen fibrils. CS/DS and keratan sulfate (KS) proteoglycans associate with collagen fibrils to maintain the characteristic collagen architecture essential for transparency of the corneal stroma (Kao et al., 2006; Hassell and Birk, 2010; Lewis et al., 2010; Quantock et al., 2010; Meek and Knupp, 2015). The anterior-most region of the corneal stroma in most species is a thin, acellular, disorganized meshwork of collagen fibrils called Bowman's layer, which is integral at its distal limit with a basement membrane that supports the corneal epithelium. An intact and properly stratified corneal epithelium is vital for clear vision. Throughout life, superficial corneal epithelial cells are constantly shed into the tear film, a loss that is counteracted by replenishment by a population of corneal epithelial stem cells at the limbus (Kinoshita et al., 2001).

## The Corneal Limbal Stem Cell Niche

**Figure 1:**
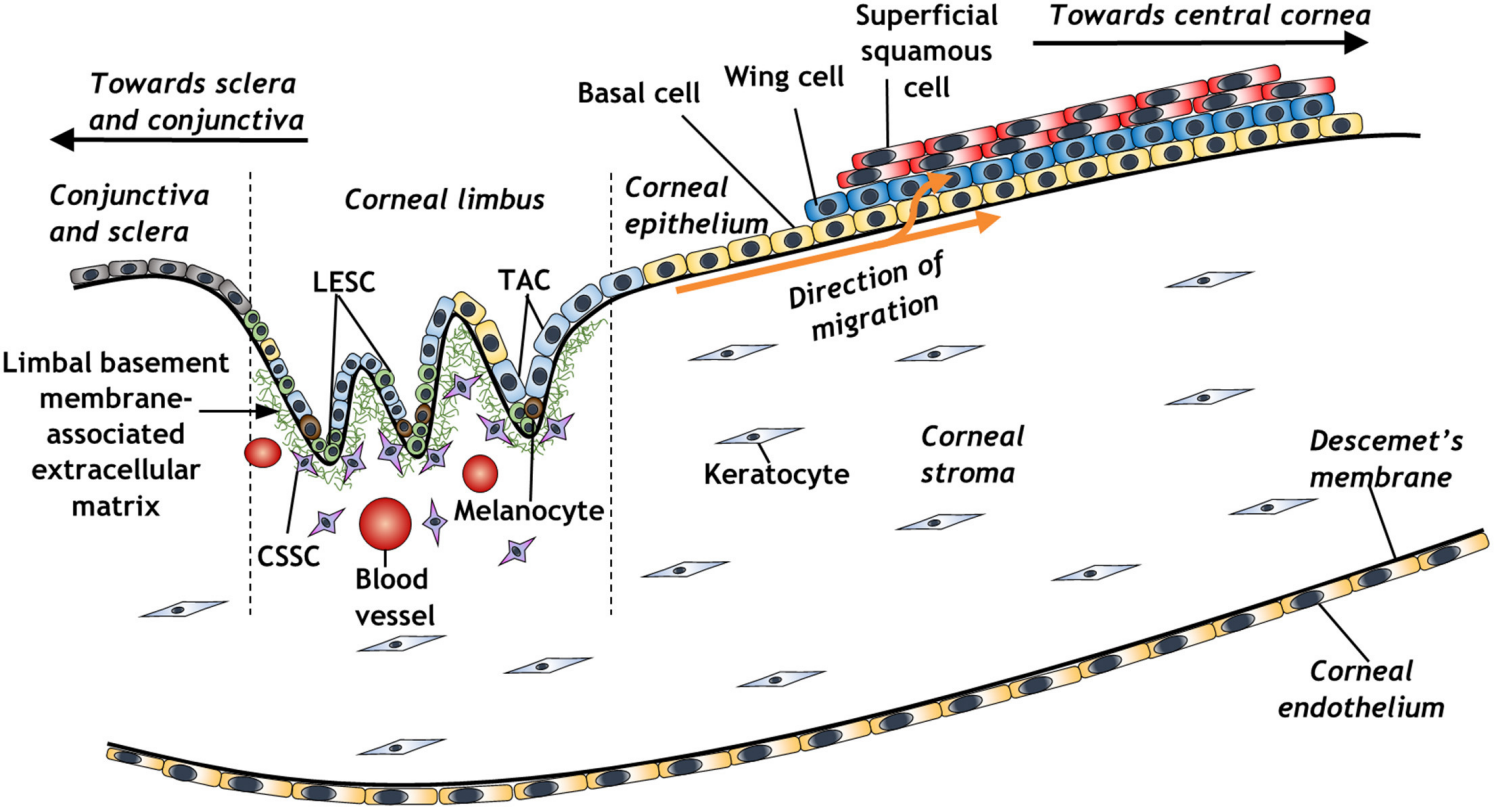
Schematic representation of the corneal limbal stem cell niche and some of its constituent elements (LESC, limbal epithelial stem cell; CSSC, corneal stromal stem cell; TAC, transient amplifying cell).

The concept of a limbal stem cell niche (Figure 1) was first proposed almost 50 years ago, as a regenerative source of epithelial cells migrating centrally from a distinct, pigmented region of the peripheral cornea (Davanger and Evensen, 1971). Further work revealed a distinct side-population of slow-cycling cells within the basal layer of corneal limbal epithelium, which appeared to be responsible for centripetal migration of epithelium and the restoration of the corneal surface in wound healing (Cotsarelis et al., 1989; Dua and Forrester, 1990). These cells were subsequently distinguished by virtue of their small size, characteristically high nucleus to cytoplasmic ratio, and expression of various stem cell markers such as ABCG2 or p63 and its various isoforms (Romano et al., 2003; Watanabe et al., 2004; Di Iorio et al., 2005; Kawakita et al., 2009). Further markers include ABCB5, which is essential for limbal epithelial stem cell maintenance and development; cells isolated using this marker are able to restore the cornea in limbal epithelial stem cell-deficient mouse models (Ksander et al., 2014). A 3D structural analysis has further elucidated the architecture of the limbal stem cell niche, revealing the presence of limbal crypts circumferentially around the eye, interspaced alongside distal invaginations of stromal extracellular matrix, termed the palisades of Vogt (Grieve et al., 2015). The identification of limbal epithelial stem cells has galvanized the study of these cells for therapeutic purposes to treat various corneal epithelial diseases (Pellegrini et al., 1997; Bains et al., 2019; Le et al., 2020).

Damage or loss of resident corneal epithelial stem cells, either through disease or injury, can lead to a limbal stem cell deficiency that ultimately results in corneal blindness, necessitating corneal surgery to restore vision. Transplantation is one treatment option for late-stage corneal pathology, however, this carries the risk of tissue rejection. Moreover, there is a distinct lack of donor tissue, with only one cornea available for every 70 required for transplantation worldwide (Gain et al., 2016). For this reason, other therapies have been investigated, including *ex vivo* expanded limbal epithelial stem cell transplantation (autograft or allograft), and the generation of an epithelial multilayer derived from oral mucosal epithelium (Oie and Nishida, 2016; Bains et al., 2019), or induced pluripotent stem cells (Hayashi et al., 2016, 2017). Whilst these pioneering technologies have shown great clinical promise, they could be further optimized by careful manipulation of culture conditions for these regenerative cells, as well as through their selection. A further potential avenue of exploration from a tissue engineering standpoint might be recreating an extracellular matrix microenvironment of the limbal stem cell niche seeded with isolated corneal limbal epithelial stem cells or induced pluripotent stem (iPS) cell derived-corneal epithelial cells.

The limbal region of the cornea also harbors a population of mesenchymal stem cells, termed corneal stromal stem cells, in the extracellular matrix subjacent to the corneal epithelial stem cell niche (Du et al., 2005). Electron microscopy has provided evidence for direct connections between corneal epithelial and stromal cells at the limbus that traverse the epithelial basement membrane (Higa et al., 2013; Dziasko et al., 2014; Yamada et al., 2015). This, along with the results of studies of the behavior of limbal epithelial and stromal cells in culture, has led to the notion of a multicellular limbal niche complex at the edge of the cornea involving both epithelial and stromal cells (Hertsenberg and Funderburgh, 2015; Dziasko and Daniels, 2016; Funderburgh et al., 2016). Work with bovine cells from the corneal stroma in culture has shown that ^35^S-labeled CS/DS, when measured by sensitivity to chondroitinase ABC, is increased 3–3.5-fold in activated fibroblasts and myofibroblasts compared with quiescent keratocytes (Funderburgh et al., 2003). To the best of our knowledge, however, the association between corneal stromal stem cells and CS has not been directly investigated. Nevertheless, it is noteworthy that the peripheral human cornea and limbus, where corneal stromal stem cells reside, contain less acidic GAG than the central cornea, primarily because KS levels are decreased (Borcherding et al., 1975). This work also indicated that chondroitin was replaced by CS at the limbus and that DS was present at detectable levels. More recently, immunohistochemistry was conducted to probe the composition of the bovine corneal stroma in which monoclonal antibody 2B6 was utilized after (i) chondroitinase ABC treatment to identify CS and DS, (ii) chondroitinase ACII treatment to identify CS, and (iii) chondroitinase B treatment to identify DS (Ho et al., 2014). This revealed that DS was present throughout the corneal stroma and into the sclera, with CS detected toward the outer periphery of the cornea and the limbus.

Investigations enabling us to accurately recreate the microenvironment of the limbal stem cell niche would be of great scientific value, not only in terms of understanding the biological functions of different components of this environment, but also because of the potential in regenerative medicine. To this end, various attempts have been made to elucidate the extracellular matrix molecules and cell-cell interactions that are important for the maintenance of the corneal limbal stem cell niche. Indeed, the corneal limbus has a distinct extracellular matrix profile compared to the central cornea and conjunctiva (Schlötzer-Schrehardt et al., 2007; Mei et al., 2012). CS, amongst other matrix molecules such as laminin isoforms and tenascin-C, are enriched at the corneal limbus where they co-localize with putative stem and progenitor cells in the basal limbal epithelium (Schlötzer-Schrehardt et al., 2007). The importance of tenascin-C in several stem cell niches has been well-documented, particularly within neural and hematopoietic environments (Seiffert et al., 1998; Garcion et al., 2001; Chiquet-Ehrismann et al., 2014). Tenascin-C, as mentioned, has been identified in the corneal limbal stem cell niche (Maseruka et al., 2000), and its spatial and temporal expression during development and wound healing, aligned to its presence in the adult limbus (Maseruka et al., 1997; Ljubimov et al., 1998; Ding et al., 2008) advocate a potential role in the self-renewal and differentiation of stem cells. It is likely that this can be achieved by providing a favorable stem cell microenvironment via interactions with other extracellular matrix components such as fibronectin (Hunt et al., 2012; Singh and Schwarzbauer, 2012) and CS, with an association between tenascin and CS having been reported in experimental models of neural repair (Gates et al., 1996).

## Chondroitin Sulfate/Dermatan Sulfate Structure and Antibodies

CS and CS/DS GAG structures are typically heterogeneous and polydisperse from molecule to molecule. The disaccharide repeat unit of CS consists of a (hex)uronic acid (glucuronic) and a hexosamine (galactosamine, typically N-acetylated), whilst in DS disaccharides the D-glucuronic acid residue is converted to αL-iduronic acid, yielding DS. Linkage of these CS disaccharide units occurs through ß3-linkage (GlcAß3GalNAc), whilst DS is through ∂3 -linkage (IdoA∂3GalNAc) (Caterson, 2012). CS/DS heterogeneity is further generated through sulfation of hydroxyl groups at positions 2, 4 and C6 on the sugar molecules, giving rise to great structural diversity (Sugahara et al., 2003; Hayes et al., 2018). Indeed, recent estimates from Persson et al. (2020) suggest, with a chain of 50 CS/DS disaccharides, an estimated 16^50^ theoretical variants arise from sixteen possible disaccharide variants. Such structural diversity is believed to be responsible for the ability of CS/DS to interact with a range of growth factors, morphogens, cytokines and chemokines, to potentially help regulate cell proliferation, cell differentiation, and tissue development (Nandini and Sugahara, 2006; Caterson, 2012; Purushothaman et al., 2012; Hayes et al., 2018; Karamanos et al., 2018). In a number of tissues, cornea included, CS exists as a co-polymer with DS forming CS/DS hybrid GAGs (Habuchi et al., 1973; Inoue and Iwasaki, 1976). An ability to recognize different sulfation patterns on CS or CS/DS is useful for the study of structurally diverse CS/DS isoforms within a tissue, and a range of monoclonal antibodies have been developed to help with this endeavor (Sorrell et al., 1990). For example, antibodies 6C3, 4C3, 7D4, and 3B3 (Caterson et al., 1985), can recognize distinct, native (i.e. non-chondroitinase-digested) and non-native (i.e., chondroitinase**-**digested) epitopes on CS/DS chains.

**Figure 2:**
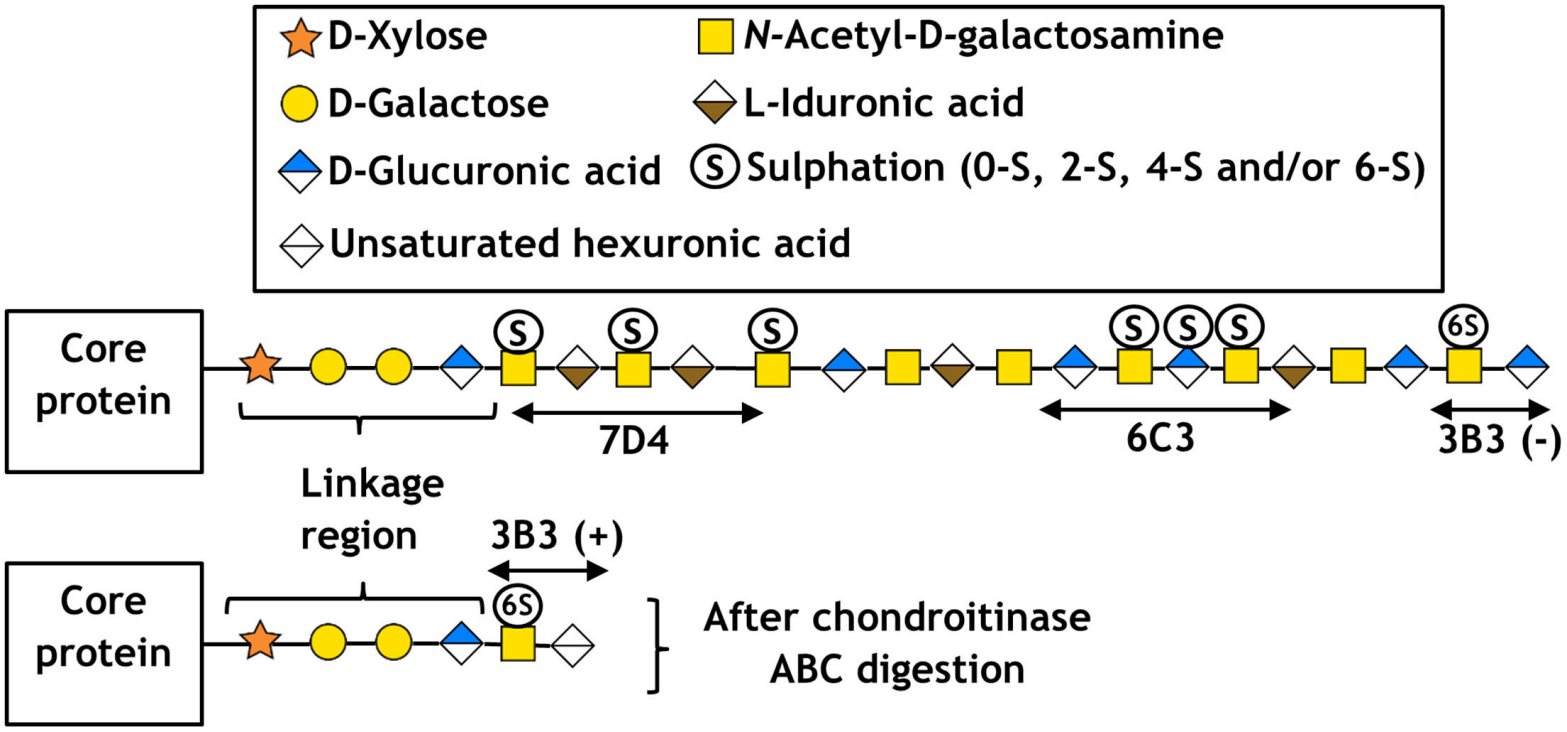
Schematic of CS/DS following the symbol nomenclature for the graphical representation of glycans (Varki et al., 2015), including the approximate location of epitopes for antibodies 7D4, 6C3, and 3B3. Respectively, (+) and (–) indicate with and without chondroitinase ABC digestion.

Some examples of these antibodies and the location of their respective CS/DS binding sites are shown in Figure 2 along with a simplified structure of a typical CS/DS hybrid chain, the location of the binding site having been deciphered by sequential enzymatic digestion. Other CS antibodies developed in our lab include 4D3 and 3B5, which also recognize a specific pattern of carbon sulfation on sequential sugar molecules within a polysaccharide chain (Sorrell et al., 1990). Whilst the exact epitope for some of these antibodies remains to be elucidated, other studies of D-type CS antibodies (which bind sites of sulfation on carbon-2 of the glucuronic acid and carbon-6 of the *N*-acetylgalactosamine sugar) have indicated they require at least an hexasaccharide (in the case of 473HD), or octasaccharide (in the case of CS-56 and MO-225), to bind and recognize a specific tetrasaccharide within the chain (Ito et al., 2005). Whilst useful, it is important to note that the flanking regions of the recognized tetrasaccharide can affect antibody affinity and *in vivo* growth factor binding. This results in overlapping oligosaccharide binding regions, termed “wobble motifs,” further increasing the complexity of epitope definition and discussed in more detail elsewhere (Caterson, 2012; Purushothaman et al., 2012).

Immunohistochemical investigations using these and other antibodies have disclosed that a number of stem cell niches exhibit specific or preferential sulfation patterns. For example, in human skin, the 6C3 epitope is located just below the basal lamina of the epidermis, adjacent to resident epidermal stem cells (Sorrell et al., 1990). This is of particular interest, given the similarities between epidermal stem cells and limbal epithelial stem cells, which both form a stratified epithelium that expresses the p63 stem cell marker (Pellegrini et al., 2001). Also, of note, is the finding that epidermal stem cells can convert into corneal epithelial-like cells when placed in corneal tissue (Gao et al., 2007). CS GAGs are also present in primordial stem cell populations in fetal tissue, such as hair bulbs and perichondrium (Hayes et al., 2016), allowing us to hypothesize a potential role for CS in maintenance of the corneal limbal stem cell niche.

## Chondroitin Sulfate in the Corneal Limbal Stem Cell Niche

Whilst much work has focused on the role of CS in the corneal stroma, less attention has been paid to a potential role for CS in the stem cell niche of the corneal limbus. The main CS proteoglycans present in cornea are decorin and biglycan, which are expressed widely throughout the corneal stroma, with biglycan levels decreasing toward maturity. CS in the cornea helps define stromal architecture, and decorin and biglycan null mice have significantly disrupted collagen ultrastructure (Zhang et al., 2009). Similar effects are seen in biglycan/lumican null mice, which display significant corneal opacity compared to mice with single proteoglycan knockouts (Chen et al., 2014). In terms of overall GAG distribution, the cornea is primarily constituted of KS GAG (both high and low sulfated forms), with CS/DS hybrid GAGs also present in slightly lower quantities, and with CS located preferentially toward the limbus (Ho et al., 2014). Heparan sulfate has also been shown to play a role in maintenance of the corneal epithelium, as its absence results in reduced corneal epithelial wound repair and improper stratification of epithelial cells (Coulson-Thomas et al., 2015).

In order to determine in more detail the specific pattern of CS distribution within the cornea, previous work by our group used the aforementioned panel of CS antibodies to map CS/DS in rabbit corneas (Yamada et al., 2015). The rabbit eye, however, differs in its microanatomy to that of human cornea and lacks a series of limbal epithelial crypts that are located circumferentially around the limbus and which form the palisades of Vogt. Limbal crypts are essentially downward focal projections of epithelial cells into the underlying extracellular matrix and run around the periphery of the cornea. The porcine limbus is believed to have a series of epithelial crypts, which is somewhat analogous to that of the human cornea. However, the positioning of the crypts around the limbal circumference of the pig eye is debated (Notara et al., 2011; Grieve et al., 2015), and new evidence about their size, shape and circumferential extent is adding to this debate (Hammond et al., unpublished results). Porcine corneas are closer in size to human corneas with a similar anatomical structure (Sanchez et al., 2011) and protein composition (Sharifi et al., 2019), thus their obvious value for corneal research. Both porcine and human corneas have a Bowman's layer – a thin, acellular layer of the distal corneal stroma immediately subjacent to the corneal epithelial basement membrane – as demonstrated by recent high-resolution investigations (Hammond et al., 2020). Bowman's layer is often missing in many other species commonly used for corneal research (Hayashi et al., 2002), identifying the pig as a reasonable animal model.

**Figure 3:**
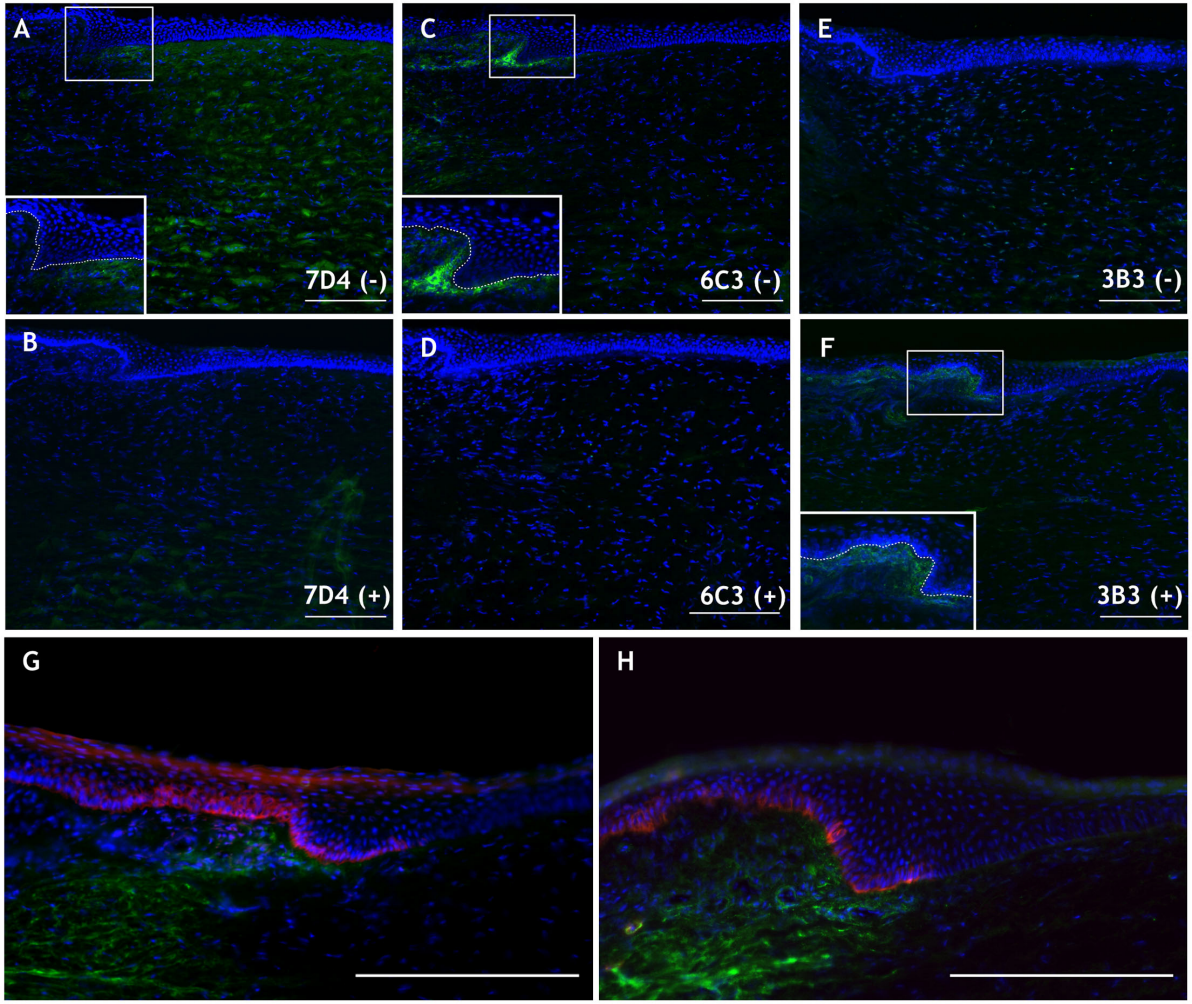
Immunohistochemical images of the peripheral porcine cornea (located to the right in each panel) and limbus. **(A,C,E)** Native 7D4, 6C3, and 3B3 epitope localization is shown in green without [i.e., (–)] chondroitinase ABC pretreatment. **(B,D,F)** Staining with the same antibodies following chondroitinase ABC treatment [i.e., (+)], which removes 7D4 and 6C3 epitopes and exposes the 3B3 epitope. Inlays in **(A,C,F)** magnify the limbal region (white box) and delineate the epithelial basement membrane (dashed line). **(G,H)** 6C3 (green) is localized in close proximity to stem cell markers ABCB5 **(G)** and keratin 19 **(H)** (both red). Scale bars = 200 μm. DAPI is employed as a nuclear stain shown in blue. Methods and negative control images with primary antibodies omitted are provided as Supplementary Material.

Native CS sulfation motifs identified by the antibodies 7D4 and 6C3 were detected in the porcine cornea (Figures 3A,C), specifically at the limbus in the case of 6C3 (Figure 3C), as was observed in the presumptive stem cell niche in rabbit cornea (Yamada et al., 2015). 3B3, an antibody recognizing non-native, chondroitinase ABC-digested stubs without digestion of a terminal disaccharide of the CS chain (6-*O*-sulfated *N*-acetylgalactosamine adjacent to the terminal glucuronate), also appeared to label CS structures surrounding the limbal niche (Figure 3F). The 6C3 CS epitope also localized in close proximity to putative limbal epithelial stem cells, as identified by two markers, ABCB5 and keratin 19 (Figures 3G,H). Although these are preliminary data, such co-localization of an extracellular matrix component and putative stem cell identifiers is an interesting discovery, which invites speculation as to a causal link in terms of the potential for the matrix to define a milieu that favorably sustains stem cell expression. With this in mind and as alluded to earlier, we also note that mesenchymal stem cells have been identified in the corneal stroma, subjacent to the basement membrane at the limbus, and are hypothesized to be supportive of the resident limbal epithelial stem cells (Du et al., 2005; Pinnamaneni and Funderburgh, 2012; Funderburgh et al., 2016). We speculate whether the 6C3 antibody might recognize cell surface-associated CS on corneal stromal stem cells or sub-epithelial stromal melanocytes that, when present within the epithelium, have been shown to be supportive of the limbal epithelial stem cell population (Dziasko et al., 2015). Ongoing studies will serve to better characterize CS in the limbal environment and aim to clarify its involvement as a potential modulator of the stem cell niche.

## Chondroitin Sulfate and Regenerative Medicine

In recent years, CS has been highlighted as essential for the functional integrity of pluripotent and multipotent stem cells, and as an important factor in the maintenance of pluripotency and differentiation propensity. For example, when CS is depleted through genetic knockout or removed by enzymatic digestion, mouse embryonic stem cells become arrested in a pluripotent state and are unable to differentiate. The addition of exogenous CS, however, recovers the differentiation propensity thus pointing to an influential role for CS in pluripotent stem cell biology (Izumikawa et al., 2014). Accordingly, CS has considerable relevance to both embryological development and stem cell biology and has been shown to be implicated in the differentiation and proliferation of multipotent stem cells from a range of tissues. Many of these studies focus on neural-derived tissue and have indicated that the removal of CS through enzymatic degradation, typically using chondroitinase ABC, can have a marked effect on neural progenitor/stem cell proliferation, differentiation and migration. The mechanism is believed to be mediated by fibroblast growth factor-2 (FGF-2) and epidermal growth factor (EGF) (Sirko et al., 2007, 2010; Gu et al., 2009). Removal of CS can also cause spontaneous differentiation of oligodendrocyte precursor cells (Karus et al., 2016), whilst the disruption of CS/DS in mesenchymal stem cells has been shown to influence osteogenic differentiation (Manton et al., 2007). Owing to the localization of specific CS motifs in corneal limbal stroma, we speculate that these molecules could potentially facilitate maintenance of corneal limbal epithelial stem cells and/or potentiate their differentiation from iPS cells *in vitro*. Various researchers have developed differentiation protocols for generating corneal epithelium from both pluripotent and multipotent stem cells. Initial attempts involved recreating the limbal niche using collagen IV and limbal fibroblast-conditioned medium to differentiate pluripotent stem cells toward a corneal epithelial phenotype. However, the cells generated were not very robust and differed from native corneal epithelium (Ahmad et al., 2007).

Other studies using pluripotent stem cells generated improved corneal epithelial-like cells that displayed corneal epithelial markers (K3/K12 and Pax-6), and showed how the regulation of *PAX6* is critical for *in vitro* differentiation (Hayashi et al., 2012; Shalom-Feuerstein et al., 2012; Brzeszczynska et al., 2014). Whilst promising, these studies failed to take into account the elaborate nature of whole eye development with the plethora of spatial and temporal developmental cues that occur between distinct cell types. One recently**-**developed method demonstrated that human iPS cells can give rise to self-forming ectodermal autonomous multi-zones (SEAMs), representing concentric zones of cells of distinct ocular lineages, including cells that resemble those of the corneal epithelium (Hayashi et al., 2016, 2017; Supplementary Video). In the original discovery of SEAM formation (Hayashi et al., 2016), which was modified by other researchers (Li et al., 2019), the cellular zones represented neuronal lineages (innermost zone 1), retina-like and neural crest-like cells (zone 2, more peripherally), ocular surface ectoderm-like cells (zone 3, more peripherally still), and finally non-ocular surface epithelial-like cells in the outermost zone 4. Lens-like cells appeared at the borders of SEAM zones 2 and 3.

**Figure 4:**
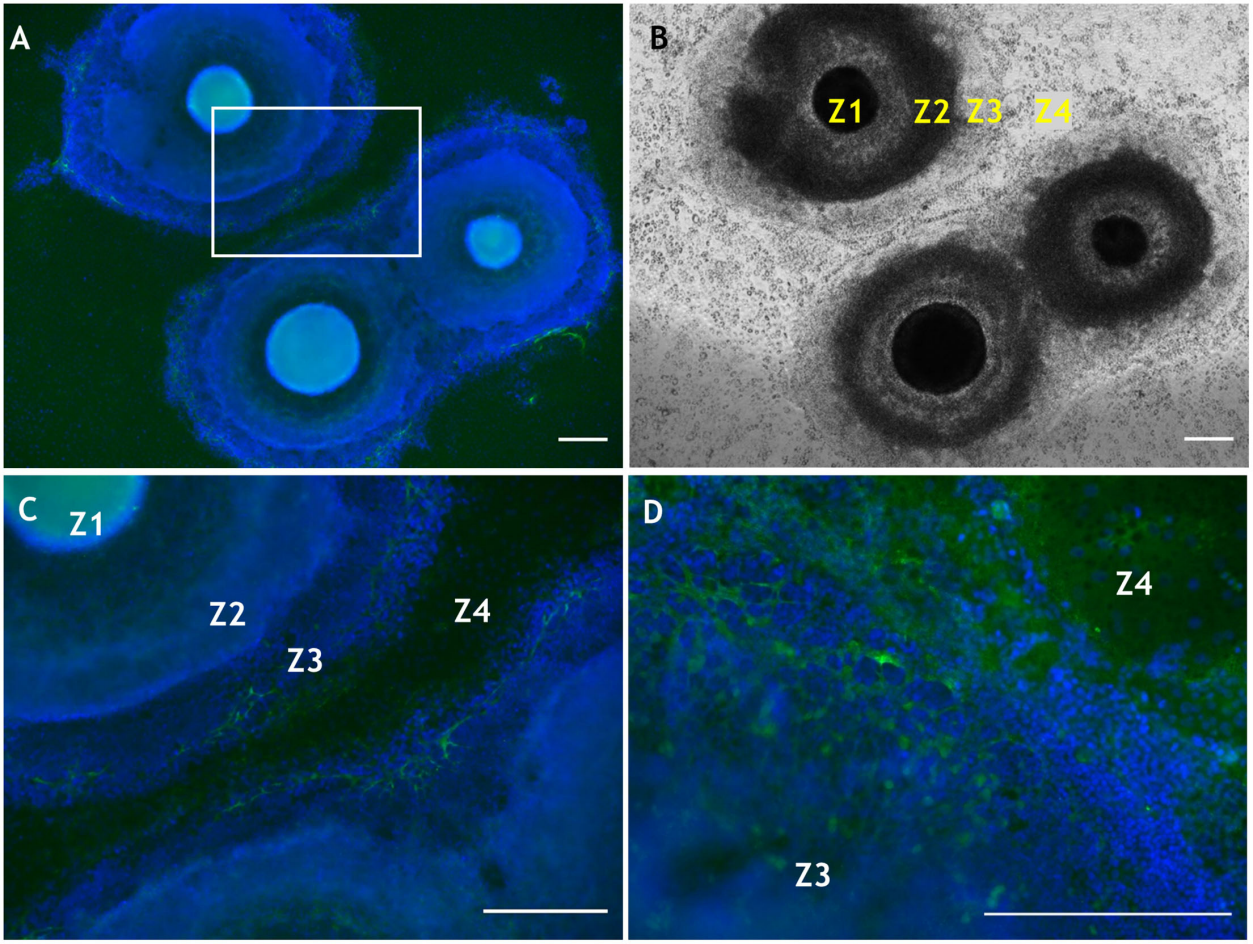
Localization of CS using antibody 7D4 within developing SEAM cultures at weeks 4 and 6 of differentiation. **(A)** Wide-field view of three SEAM colonies at week 4 of differentiation, two of which are beginning to merge (7D4 is shown in green and nuclear Hoechst-334 staining in blue). **(B)** Bright-field image of a SEAM at week 4 of differentiation with each zone (Z) indicated. **(C)** Higher magnification view of CS immunolocalisation in a selected region in **(A)**. **(D)** High magnification of a SEAM at week 6 of differentiation indicating a widespread deposition of CS. Scale bars = 100 μm. Methods and negative control images with primary antibodies omitted are provided as Supplementary Material.

Further work involving manipulation within the substrate of laminin, another extracellular matrix molecule present in basement membrane of the corneal limbus, showed that different isoforms differentially influence the differentiation propensity of corneal epithelial cells derived from human iPS cells (Shibata et al., 2018). Laminin can also influence cell phenotype based upon the selective adhesiveness of the cells toward various substrates (Shibata et al., 2020). We also note, in relation to the eye, that both CS and laminin are temporally and spatially expressed during optic nerve regeneration in fish (Battisti et al., 1992), and more recently have been applied in the expansion of corneal endothelial cells for potential transplantation (Kennedy et al., 2019). In addition, CS and laminin are suggested to have counteractive roles in precursor cell differentiation, due to regulation of the β1-integrin signaling pathway (Sun et al., 2017), which highlights potential impacting interactions (indirect and/or direct) between these ECM molecules. As mentioned, laminin influences SEAM development, and some recent preliminary data indicate that CS, too, is likely involved in the differentiation of human iPS cells in a developing SEAM as it is increasingly deposited from weeks 4–6 (Figure 4). Specifically, the CS moiety revealed by 7D4 was detected in SEAM zones 3, outward to zone 4 (Figures 4A–C). Furthermore, CS is ubiquitously deposited into the surrounding matrix by week 6 (Figure 4D). Thus, facilitating deposition or exogenous supply of CS, could potentially be used to modulate differentiation of human iPS cells in SEAMs.

Whilst the application of CS to bioengineered scaffolds and its use to promote cell differentiation is not new, there are few investigations into defined species of CS and their influence on stem cell differentiation in the context of the corneal epithelium. Also, seldom considered is the fact that commercially prepared CS can often contain significant impurities, for example KS (Santos et al., 2017). Essentially, there has been relatively little detailed exploration into possible modulatory roles for CS, or indeed other GAGs, within the corneal limbal stem cell niche, but emerging knowledge will provide an enhanced understanding of the influence of CS upon the behavior of human stem cells and their potential involvement in corneal regenerative medicine.

## Data Availability Statement

The original contributions presented in the study are included in the article/Supplementary Materials, further inquiries can be directed to the corresponding author/s.

## Author Contributions

AQ, SA, and CH planned the article. SA composed Figures 1, 2. GH and SA obtained the data presented in Figure 3. JH, SA, and RH obtained the data presented in Figure 4. AQ, JRe, JRa, CH, RH, and KN obtained funding. All authors contributed to the writing of the article.

## Conflict of Interest

The authors declare that the research was conducted in the absence of any commercial or financial relationships that could be construed as a potential conflict of interest.

## References

[B1] Ahmad S. Stewart R. Yung S. Kolli S. Armstrong L. Stojkovic M.. (2007). Differentiation of human embryonic stem cells into corneal epithelial-like cells by *in vitro* replication of the corneal epithelial stem cell niche. Stem Cells 25, 1145–1155. 10.1634/stemcells.2006-0516 17255521

[B2] Bains K. K. Fukuoka H. Hammond G. M. Sotozono C. Quantock A. J. (2019). Recovering vision in corneal epithelial stem cell deficient eyes. Cont. Lens Anterior Eye 42, 350–358. 10.1016/j.clae.2019.04.006 31047800 PMC6611221

[B3] Battisti W. P. Shinar Y. Schwartz M. Levitt P. Murray M. (1992). Temporal and spatial patterns of expression of laminin, chondroitin sulphate proteoglycan and HNK-1 immunoreactivity during regeneration in the goldfish optic nerve. J. Neurocytol. 21, 557–573. 10.1002/cne.903540211 1380544

[B4] Borcherding M. S. Blacik L. J. Sittig R. A. Bizzell J. W. Breen M. Weinstein H. G. (1975). Proteoglycans and collagen fibre organization in human corneoscleral tissue. Exp. Eye Res. 21, 59–70. 10.1016/0014-4835(75)90057-3 124659

[B5] Brzeszczynska J. Samuel K. Greenhough S. Ramaesh K. Dhillon B. Hay D. C.. (2014). Differentiation and molecular profiling of human embryonic stem cell-derived corneal epithelial cells. Int. J. Mol. Med. 33, 1597–1606. 10.3892/ijmm.2014.1714 24676408

[B6] Caterson B. (2012). Fell-muir lecture: chondroitin sulphate glycosaminoglycans: fun for some and confusion for others. Int. J. Exp. Pathol. 93, 1–10. 10.1111/j.1365-2613.2011.00807.x 22264297 PMC3311016

[B7] Caterson B. Christner J. E. Baker J. R. Couchman J. R. (1985). Production and characterization of monoclonal antibodies directed against connective tissue proteoglycans. *Fed*. Proc. 44, 386–393. 2578417

[B8] Chen S. Birk D. E. (2011). Focus on molecules: decorin. Exp. Eye Res. 92, 444–445. 10.1016/j.exer.2010.05.008 20493188 PMC2957536

[B9] Chen S. Mienaltowski M. J. Birk D. E. (2015). Regulation of corneal stroma extracellular matrix assembly. Exp. Eye Res. 133, 69–80. 10.1016/j.exer.2014.08.001 25819456 PMC4379422

[B10] Chen S. Young M. F. Chakravarti S. Birk D. E. (2014). Interclass small leucine-rich repeat proteoglycan interactions regulate collagen fibrillogenesis and corneal stromal assembly. Matrix Biol. 35, 103–111. 10.1016/j.matbio.2014.01.004 24447998 PMC4039618

[B11] Chiquet-Ehrismann R. Orend G. Chiquet M. Tucker R. P. Midwood K. S. (2014). Tenascins in stem cell niches. Matrix Biol. 37, 112–123. 10.1016/j.matbio.2014.01.007 24472737

[B12] Cotsarelis G. Cheng S. Z. Dong G. Sun T. T. Lavker R. M. (1989). Existence of slow-cycling limbal epithelial basal cells that can be preferentially stimulated to proliferate: implications on epithelial stem cells. Cell 57, 201–209. 10.1016/0092-8674(89)90958-6 2702690

[B13] Coulson-Thomas V. J. Chang S.-H. Yeh L.-K. Coulson-Thomas Y. M. Yamaguchi Y. Esko J.. (2015). Loss of corneal epithelial heparan sulfate leads to corneal degeneration and impaired wound healing. Invest. Ophthalmol. Vis. Sci. 56, 3004–3014. 10.1167/iovs.14-15341 26024086 PMC4432551

[B14] Davanger M. Evensen A. (1971). Role of the pericorneal papillary structure in renewal of corneal epithelium. Nature 229, 560–561. 10.1038/229560a0 4925352

[B15] Di Iorio E. Barbaro V. Ruzza A. Ponzin D. Pellegrini G. De Luca M. (2005). Isoforms of DeltaNp63 and the migration of ocular limbal cells in human corneal regeneration. Proc. Natl. Acad. Sci. U.S.A. 102, 9523–9528. 10.1073/pnas.0503437102 15983386 PMC1172259

[B16] Ding Z. Dong J. Liu J. Deng S. X. (2008). Preferential gene expression in the limbus of the vervet monkey. Mol Vis. 14, 2031–2041. 18989386 PMC2579939

[B17] Du Y. Funderburgh M. L. Mann M. M. SundarRaj N. Funderburgh J. L. (2005). Multipotent stem cells in human corneal stroma. Stem Cells 23, 1266–1275. 10.1634/stemcells.2004-0256 16051989 PMC1941788

[B18] Dua H. S. Forrester J. V. (1990). The corneoscleral limbus in human corneal epithelial wound healing. Am. J. Ophthalmol. 110, 646–656. 10.1016/s0002-9394(14)77062-x 2248329

[B19] Dziasko M. A. Armer H. E. Levis H. J. Shortt A. J. Tuft S. Daniels J. T. (2014). Localisation of epithelial cells capable of holoclone formation *in vitro* and direct interaction with stromal cells in the native human limbal crypt. PLoS ONE 9:e94283. 10.1371/journal.pone.0094283 24714106 PMC3979808

[B20] Dziasko M. A. Daniels J. T. (2016). Anatomical features and cell-cell interactions in the human limbal epithelial stem cell niche. Ocul. Surf. 14, 322–330. 10.1016/j.jtos.2016.04.002 27151422

[B21] Dziasko M. A. Tuft S. J. Daniels J. T. (2015). Limbal melanocytes support limbal epithelial stem cells in 2D and 3D microenvironments. Exp. Eye Res. 138, 70–79. 10.1016/j.exer.2015.06.026 26142953

[B22] Farrugia B. L. Lord M. S. Whitelock J. M. Melrose J. (2018). Harnessing chondroitin sulphate in composite scaffolds to direct progenitor and stem cell function for tissue repair. Biomater. Sci. 6, 947–957. 10.1039/C7BM01158J 29560990

[B23] Funderburgh J. L. Funderburgh M. L. Du Y. (2016). Stem cells in the limbal stroma. Ocul. Surf. 14, 113–120. 10.1016/j.jtos.2015.12.006 26804252 PMC4842326

[B24] Funderburgh J. L. Mann M. M. Funderburgh M. L. (2003). Keratocyte phenotype mediates proteoglycan structure: a role for fibroblasts in corneal fibrosis. J. Biol. Chem. 278, 45629–45637. 10.1074/jbc.M303292200 12933807 PMC2877919

[B25] Gain P. Jullienne R. He Z. Aldossary M. Acquart S. Cognasse F.. (2016). Global survey of corneal transplantation and eye banking. JAMA Ophthalmol. 134, 167–173. 10.1001/jamaophthalmol.2015.4776 26633035

[B26] Gao N. Wang Z. Huang B. Ge J. Lu R. Zhang K.. (2007). Putative epidermal stem cell convert into corneal epithelium-like cell under corneal tissue *in vitro*. Sci. China C Life Sci. 50, 101–110. 10.1007/s11427-007-0006-4 17393090

[B27] Garcion E. Faissner A. ffrench-Constant C. (2001). Knockout mice reveal a contribution of the extracellular matrix molecule tenascin-C to neural precursor proliferation and migration. Development 128, 2485–2496. Available online at: https://dev.biologists.org/content/128/13/2485 11493565 10.1242/dev.128.13.2485

[B28] Gates M. A. Fillmore H. Steindler D. A. (1996). Chondroitin sulfate proteoglycan and tenascin in the wounded adult mouse neostriatum *in vitro*: dopamine neuron attachment and process outgrowth. J. Neurosci. 16, 8005–8018. 10.1523/JNEUROSCI.16-24-08005.1996 8987827 PMC6579225

[B29] Grieve K. Ghoubay D. Georgeon C. Thouvenin O. Bouheraoua N. Paques M.. (2015). Three-dimensional structure of the mammalian limbal stem cell niche. Exp. Eye Res. 140, 75–84. 10.1016/j.exer.2015.08.003 26297801

[B30] Gu W. L. Fu S. L. Wang Y. X. Li Y. Lu H. Z. Xu X. M.. (2009). Chondroitin sulfate proteoglycans regulate the growth, differentiation and migration of multipotent neural precursor cells through the integrin signaling pathway. BMC Neurosci. 10:128. 10.1186/1471-2202-10-128 19845964 PMC2773784

[B31] Habuchi H. Yamagata T. Iwata H. Suzuki S. (1973). The occurrence of a wide variety of dermatan sulfate-chondroitin sulfate copolymers in fibrous cartilage. J. Biol. Chem. 248, 6019–6028. 4269443

[B32] Hammond G. M. Young R. D. Muir D. D. Quantock A. J. (2020). The microanatomy of Bowman's layer in the cornea of the pig: changes in collagen fibril structure at the corneoscleral limbus. Eur. J. Anat. 24, 399–406. Available online at: http://www.eurjanat.com/web/paper.php?id=200195gh

[B33] Hassell J. R. Birk D. E. (2010). The molecular basis of corneal transparency. Exp. Eye Res. 91, 326–335. 10.1016/j.exer.2010.06.021 20599432 PMC3726544

[B34] Hayashi R. Ishikawa Y. Ito M. Kageyama T. Takashiba K. Fujioka T.. (2012). Generation of corneal epithelial cells from induced pluripotent stem cells derived from human dermal fibroblast and corneal limbal epithelium. PLoS ONE 7:e45435. 10.1371/journal.pone.0045435 23029008 PMC3454439

[B35] Hayashi R. Ishikawa Y. Katori R. Sasamoto Y. Taniwaki Y. Takayanagi H.. (2017). Coordinated generation of multiple ocular-like cell lineages and fabrication of functional corneal epithelial cell sheets from human iPS cells. Nat. Protoc. 12, 683–696. 10.1038/nprot.2017.007 28253236

[B36] Hayashi R. Ishikawa Y. Sasamoto Y. Katori R. Nomura N. Ichikawa T.. (2016). Co-ordinated ocular development from human iPS cells and recovery of corneal function. Nature 531, 376–380. 10.1038/nature17000 26958835

[B37] Hayashi S. Osawa T. Tohyama K. (2002). Comparative observations on corneas, with special reference to Bowman's layer and Descemet's membrane in mammals and amphibians. J. Morphol. 254, 247–258. 10.1002/jmor.10030 12386895

[B38] Hayes A. Sugahara K. Farrugia B. Whitelock J. M. Caterson B. Melrose J. (2018). Biodiversity of CS–proteoglycan sulphation motifs: chemical messenger recognition modules with roles in information transfer, control of cellular behaviour and tissue morphogenesis. Biochem. J. 475, 587–620. 10.1042/BCJ20170820 29439148

[B39] Hayes A. J. Hughes C. E. Smith S. M. Caterson B. Little C. B. Melrose J. (2016). The CS sulfation motifs 4C3, 7D4, 3B3[–]; and perlecan identify stem cell populations and their niches, activated progenitor cells and transitional areas of tissue development in the fetal human elbow. Stem Cells Dev. 25, 836–847. 10.1089/scd.2016.0054 27068010

[B40] Hayes A. J. Tudor D. Nowell M. A. Caterson B. Hughes C. E. (2008). Chondroitin sulfate sulfation motifs as putative biomarkers for isolation of articular cartilage progenitor cells. J. Histochem. Cytochem. 56, 125–138. 10.1369/jhc.7A7320.2007 17938280 PMC2324172

[B41] Hertsenberg A. J. Funderburgh J. L. (2015). Stem cells in the cornea. Prog. Mol. Biol. Transl. Sci. 134, 25–41. 10.1016/bs.pmbts.2015.04.002 26310147 PMC5327505

[B42] Higa K. Kato N. Yoshida S. Ogawa Y. Shimazaki J. Tsubota K.. (2013). Aquaporin 1-positive stromal niche-like cells directly interact with N-cadherin-positive clusters in the basal limbal epithelium. Stem Cell Res. 10, 147–155. 10.1016/j.scr.2012.11.001 23276695

[B43] Ho L. T. Y. Harris A. M. Tanioka H. Yagi N. Kinoshita S. Caterson B.. (2014). A comparison of glycosaminoglycan distributions, keratan sulphate sulphation patterns and collagen fibril architecture from central to peripheral regions of the bovine cornea. Matrix Biol. 38, 59–68. 10.1016/j.matbio.2014.06.004 25019467 PMC4199143

[B44] Hunt G. C. Singh P. Schwarzbauer J. E. (2012). Endogenous production of fibronectin is required for self-renewal of cultured mouse embryonic stem cells. Exp. Cell Res. 318, 1820–1831. 10.1016/j.yexcr.2012.06.009 22710062 PMC3582329

[B45] Inoue S. Iwasaki M. (1976). Dermatan sulfate-chondroitin sulfate copolymers from ambilical cord isolation and characterization. J. Biochem. 80, 513–524. 10.1093/oxfordjournals.jbchem.a131306 977551

[B46] Ito Y. Hikino M. Yajima Y. Mikami T. Sirko S. von Holst A.. (2005). Structural characterization of the epitopes of the monoclonal antibodies 473HD, CS-56, and MO-225 specific for chondroitin sulfate D-type using the oligosaccharide library. Glycobiology 15, 593–603. 10.1093/glycob/cwi036 15625183

[B47] Izumikawa T. Sato B. Kitagawa H. (2014). Chondroitin sulfate is indispensable for pluripotency and differentiation of mouse embryonic stem cells. Sci. Rep. 4:3701. 10.1038/srep03701 24424429 PMC3892716

[B48] Kao W. W. Funderburgh J. L. Xia Y. Liu C. Y. Conrad G. W. (2006). Focus on molecules: lumican. Exp. Eye Res. 82, 3–4. 10.1016/j.exer.2005.08.012 16213485 PMC2876311

[B49] Karamanos N. K. Piperigkou Z. Theocharis A. D. Watanabe H. Franchi M. Baud S.. (2018). Proteoglycan chemical diversity drives multifunctional cell regulation and therapeutics. Chem. Rev. 118, 9152–9232. 10.1021/acs.chemrev.8b00354 30204432

[B50] Karus M. Ulc A. Ehrlich M. Czopka T. Hennen E. Fischer J.. (2016). Regulation of oligodendrocyte precursor maintenance by chondroitin sulphate glycosaminoglycans. Glia 64, 270–286. 10.1002/glia.22928 26454153

[B51] Kawakita T. Shimmura S. Higa K. Espana E. M. He H. Shimazaki J.. (2009). Greater growth potential of p63-positive epithelial cell clusters maintained in human limbal epithelial sheets. Invest. Ophthalmol. Vis. Sci. 50, 4611–4617. 10.1167/iovs.08-2586 19324845 PMC2846109

[B52] Kennedy S. Lace R. Carserides C. Gallagher A. G. Wellings D. A. Williams R. L.. (2019). Poly-ε-lysine based hydrogels as synthetic substrates for the expansion of corneal endothelial cells for transplantation. J. Mater. Sci. Mater. Med. 30:102. 10.1007/s10856-019-6303-1 31485761 PMC6726667

[B53] Kinoshita S. Adachi W. Sotozono C. Nishida K. Yokoi N. Quantock A. J.. (2001). Characteristics of the human ocular surface epithelium. Prog. Retin. Eye Res. 20, 639–673. 10.1016/s1350-9462(01)00007-6 11470454

[B54] Ksander B. R. Kolovou P. E. Wilson B. J. Saab K. R. Guo Q. Ma J.. (2014). ABCB5 is a limbal stem cell gene required for corneal development and repair. Nature 511, 353–357. 10.1038/nature13426 25030174 PMC4246512

[B55] Le Q. Chauhan T. Yung M. Tseng C. H. Deng S. X. (2020). Outcomes of limbal stem cell transplant: a meta-analysis. JAMA Ophthalmol. 138, 660–670. 10.1001/jamaophthalmol.2020.1120 32324211 PMC7180742

[B56] Lewis P. N. Pinali C. Young R. D. Meek K. M. Quantock A. J. Knupp C. (2010). Structural interactions between collagen and proteoglycans are elucidated by three-dimensional electron tomography of bovine cornea. Structure 18, 239–245. 10.1016/j.str.2009.11.013 20159468

[B57] Li Z. Duan H. Li W. Hu X. Jia Y. Zhao C.. (2019). Rapid differentiation of multi-zone ocular cells from human induced pluripotent stem cells and generation of corneal epithelial and endothelial cells. Stem Cells Dev. 28, 454–463. 10.1089/scd.2018.0176 30712489

[B58] Ljubimov A. V. Saghizadeh M. Spirin K. S. Mecham R. P. Sakai L. Y. Kenney M. C. (1998). Increased expression of fibrillin-1 in human corneas with bullous keratopathy. Cornea 17, 309–314. 9603388

[B59] Manton K. J. Leong D. F. M. Cool S. M. Nurcombe V. (2007). Disruption of heparan and chondroitin sulfate signaling enhances mesenchymal stem cell-derived osteogenic differentiation via bone morphogenetic protein signaling pathways'. Stem Cells 25, 2845–2854. 10.1634/stemcells.2007-0065 17702986

[B60] Maseruka H. Bonshek R. E. Tullo A. B. (1997). Tenascin-C expression in normal, inflamed, and scarred human corneas. Br. J. Ophthalmol. 81, 677–682. 10.1136/bjo.81.8.677 9349157 PMC1722281

[B61] Maseruka H. Ridgway A. Tullo A. Bonshek R. (2000). Developmental changes in patterns of expression of tenascin-C variants in the human cornea. Invest. Ophthalmol. Vis. Sci. 41, 4101–4107. Available online at: https://iovs.arvojournals.org/article.aspx?articleid=2162675 11095602

[B62] Meek K. M. Knupp C. (2015). Corneal structure and transparency. Prog. Retin. Eye Res. 49, 1–16. 10.1016/j.preteyeres.2015.07.001 26145225 PMC4655862

[B63] Mei H. Gonzalez S. Deng S. X. (2012). Extracellular matrix is an important component of limbal stem cell niche. J. Funct. Biomater. 3, 879–894. 10.3390/jfb3040879 24955751 PMC4030928

[B64] Melrose J. Isaacs M. D. Smith S. M. Hughes C. E. Little C. B. Caterson B.. (2012). Chondroitin sulphate and heparan sulphate sulphation motifs and their proteoglycans are involved in articular cartilage formation during human foetal knee joint development. Histochem. Cell Biol. 138, 461–475. 10.1007/s00418-012-0968-6 22617995

[B65] Nandini C. D. Sugahara K. (2006). Role of the sulfation pattern of chondroitin sulfate in its biological activities and in the binding of growth factors. Adv. Pharmacol. 53, 253–279. 10.1016/S1054-3589(05)53012-6 17239770

[B66] Notara M. Schrader S. Daniels J. T. (2011). The porcine limbal epithelial stem cell niche as a new model for the study of transplanted tissue-engineered human limbal epithelial cells. Tissue Eng. Part A 17, 741–750. 10.1089/ten.TEA.2010.0343 20929285

[B67] Oie Y. Nishida K. (2016). Corneal regenerative medicine. Regen. Ther. 5, 40–45. 10.1016/j.reth.2016.06.002 31245499 PMC6581846

[B68] Parfitt G. J. Pinali C. Akama T. O. Young R. D. Nishida K. Quantock A. J.. (2011). Electron tomography reveals multiple self-association of chondroitin sulphate/dermatan sulphate proteoglycans in Chst5-null mouse corneas. J. Struct. Biol. 174, 536–541. 10.1016/j.jsb.2011.03.015 21440637

[B69] Pellegrini G. Dellambra E. Golisano O. Martinelli E. Fantozzi I. Bondanza S.. (2001). P63 identifies keratinocyte stem cells. Proc. Natl. Acad. Sci. U.S.A. 98, 3156–3161. 10.1073/pnas.061032098 11248048 PMC30623

[B70] Pellegrini G. Traverso C. E. Franzi A. T. Zingirian M. Cancedda R. De Luca M. (1997). Long-term restoration of damaged corneal surfaces with autologous cultivated corneal epithelium. Lancet 349, 990–993. 10.1016/S0140-6736(96)11188-0 9100626

[B71] Persson A. Vorontsov E. Larson G. Nilsson J. (2020). Glycosaminoglycan domain mapping of cellular chondroitin/dermatan sulfates. Sci. Rep. 10:3506. 10.1038/s41598-020-60526-0 32103093 PMC7044218

[B72] Pinnamaneni N. Funderburgh J. L. (2012). Concise review: stem cells in the corneal stroma. Stem Cells 30, 1059–1063. 10.1002/stem.1100 22489057 PMC3580383

[B73] Purushothaman A. Sugahara K. Faissner A. (2012). Chondroitin sulfate “wobble motifs” modulate maintenance and differentiation of neural stem cells and their progeny. J. Biol. Chem. 287, 2935–2942. 10.1074/jbc.R111.298430 22094467 PMC3270950

[B74] Quantock A. J. Young R. D. Akama T. O. (2010). Structural and biochemical aspects of keratan sulphate in the cornea. Cell. Mol. Life Sci. 67, 891–906. 10.1007/s00018-009-0228-7 20213925 PMC11115788

[B75] Romano A. C. Espana E. M. Yoo S. H. Budak M. T. Wolosin J. M. Tseng S. C. G. (2003). Different cell sizes in human limbal and central corneal basal epithelia measured by confocal microscopy and flow cytometry. Invest. Ophthalmol. Vis. Sci. 44, 5125–5129. 10.1167/iovs.03-0628 14638707

[B76] Sanchez I. Martin R. Ussa F. Fernandez-Bueno I. (2011). The parameters of the porcine eyeball. Graefes Arch. Clin. Exp. Ophthalmol. 249, 475–482. 10.1007/s00417-011-1617-9 21287191

[B77] Santos G. R. C. Piquet A. A. Glauser B. F. Tovar A. M. F. Pereira M. S. Vilanova E.. (2017). Systematic analysis of pharmaceutical preparations of chondroitin sulfate combined with glucosamine. Pharmaceuticals 10:38. 10.3390/ph10020038 28368296 PMC5490395

[B78] Schlötzer-Schrehardt U. Dietrich T. Saito K. Sorokin L. Sasaki T. Paulsson M.. (2007). Characterization of extracellular matrix components in the limbal epithelial stem cell compartment. Exp. Eye Res. 85, 845–860. 10.1016/j.exer.2007.08.020 17927980

[B79] Seiffert M. Beck S. C. Schermutzki F. Müller C. A. Erickson H. P. Klein G. (1998). Mitogenic and adhesive effects of tenascin-C on human hematopoietic cells are mediated by various functional domains. Matrix Biol. 17, 47–63. 10.1016/s0945-053x(98)90124-x 9628252

[B80] Shalom-Feuerstein R. Serror L. De La Forest Divonne S. Petit I. Aberdam E. Camargo L.. (2012). Pluripotent stem cell model reveals essential roles for miR-450b-5p and miR-184 in embryonic corneal lineage specification. Stem Cells 30, 898–909. 10.1002/stem.1068 22367714

[B81] Sharifi R. Yang Y. Adibnia Y. Dohlman C. H. Chodosh J. Gonzalez-Andrades M. (2019). Finding an optimal corneal xenograft using comparative analysis of corneal matrix proteins across species. Sci. Rep. 9:1876. 10.1038/s41598-018-38342-4 30755666 PMC6372616

[B82] Shibata S. Hayashi R. Kudo Y. Okubo T. Imaizumi T. Katayama T.. (2020). Cell-type-specific adhesiveness and proliferation propensity on laminin isoforms enable purification of iPSC-derived corneal epithelium. Stem Cell Rep. 14, 663–676. 10.1016/j.stemcr.2020.02.008 32197114 PMC7160305

[B83] Shibata S. Hayashi R. Okubo T. Kudo Y. Katayama T. Ishikawa Y.. (2018). Selective laminin-directed differentiation of human induced pluripotent stem cells into distinct ocular lineages. Cell Rep. 25, 1668–1679. 10.1016/j.celrep.2018.10.032 30404017

[B84] Singh P. Schwarzbauer J. E. (2012). Fibronectin and stem cell differentiation - lessons from chondrogenesis. J. Cell Sci. 125, 3703–3712. 10.1242/jcs.095786 22976308 PMC3462078

[B85] Sirko S. von Holst A. Weber A. Wizenmann A. Theocharidis U. Götz M.. (2010). Chondroitin sulfates are required for fibroblast growth factor-2-dependent proliferation and maintenance in neural stem cells and for epidermal growth factor-dependent migration of their progeny. Stem Cells 28, 775–787. 10.1002/stem.309 20087964

[B86] Sirko S. von Holst A. Wizenmann A. Götz M. Faissner A. (2007). Chondroitin sulfate glycosaminoglycans control proliferation, radial glia cell differentiation and neurogenesis in neural stem/progenitor cells. Development 134, 2727–2738. 10.1242/dev.02871 17596283

[B87] Sorrell J. M. Mahmoodian F. Schafer I. A. Davis B. Caterson B. (1990). Identification of monoclonal antibodies that recognize novel epitopes in native chondroitin/dermatan sulfate glycosaminoglycan chains: their use in mapping functionally distinct domains of human skin. J. Histochem. Cytochem. 38, 393–402. 10.1177/38.3.1689338 1689338

[B88] Sugahara K. Mikami T. Uyama T. Mizuguchi S. Nomura K. Kitagawa H. (2003). Recent advances in the structural biology of chondroitin sulfate and dermatan sulfate. Curr. Opin. Struct. Biol. 13, 612–620. 10.1016/j.sbi.2003.09.011 14568617

[B89] Sun Y. Deng Y. Xiao M. Hu L. Li Z. Chen C. (2017). Chondroitin sulfate proteoglycans inhibit the migration and differentiation of oligodendrocyte precursor cells and its counteractive interaction with laminin. Int. J. Mol. Med. 40, 1657–1668. 10.3892/ijmm.2017.3153 29039438 PMC5716457

[B90] Varki A. Cummings R. D. Aebi M. Packer N. H. Seeberger P. H. Esko J. D.. (2015). Symbol nomenclature for graphical representations of glycans. Glycobiology 25, 1323–1324. 10.1093/glycob/cwv091 26543186 PMC4643639

[B91] Watanabe K. Nishida K. Yamato M. Umemoto T. Sumide T. Yamamoto K.. (2004). Human limbal epithelium contains side population cells expressing the ATP-binding cassette transporter ABCG2. FEBS Lett. 565, 6–10. 10.1016/j.febslet.2004.03.064 15135043

[B92] Yamada K. Young R. D. Lewis P. N. Shinomiya K. Meek K. M. Kinoshita S.. (2015). Mesenchymal–epithelial cell interactions and proteoglycan matrix composition in the presumptive stem cell niche of the rabbit corneal limbus. Mol. Vis. 21, 1328–1339. 26788025 PMC4704773

[B93] Zhang G. Chen S. Goldoni S. Calder B. W. Simpson H. C. Owens R. T.. (2009). Genetic evidence for the coordinated regulation of collagen fibrillogenesis in the cornea by decorin and biglycan. J. Biol. Chem. 284, 8888–8897. 10.1074/jbc.M806590200 19136671 PMC2659246

