# Supplementary material for: Chondroitin Sulfate as a Potential Modulator of the Stem Cell Niche in Cornea
Source: Front Cell Dev Biol. 2021 Jan 12;8:567358. doi: 10.3389/fcell.2020.567358 (PMC7835413; doi:10.3389/fcell.2020.567358)
Supplement: Supplementary file 1 [file Data_Sheet_1.docx]

**SUPPLEMENTARY MATERIAL**

**METHODS AND NEGATIVE CONTROL IMAGES OF ANTIBODY STAINING**

**Immunohistochemistry of Porcine Cornea**

Porcine cornea samples frozen in OCT were cryosectioned at 15 µm thickness and adhered to Superfrost glass slides (Thermofisher, Loughborough) prior to staining. All sections were rehydrated in PBS for 5 min, before non-specific staining was blocked by immersion in PBS with 1% bovine serum albumin (BSA) and 0.05% Tween 20, for 1 hour at room temperature. All primary and secondary antibodies were diluted in PBS with 1% BSA and 0.05% Tween 20. Anti-CS primary antibody concentrations were first optimised and then applied as follows: 6C3 (1 in 10 dilution, mouse IgM), 7D4 (1 in 100 dilution, mouse IgM), 4C3 (1 in 100 dilution, mouse IgM) (Sorrell et al., 1990), all for approximately 16 hours at 4°C. For dual localisation of CS and putative stem cell markers, primary antibodies for ABCB5 (NBP1-77687, 1:100, Novus) and Cytokeratin 19 (EP1580Y, 1:100, Abcam) were included with the 6C3 antibody. To validate anti-CS antibody staining, some sections were pre-digested with chondroitinase ABC enzyme to remove epitopes on CS chains, as follows. Sections were rehydrated in PBS for 5 min and then incubated for 2 hours at 37°C in 0.1 U/ml chondroitinase ABC (Sigma-Aldrich, Gillingham) in 50mM Tris-HCl buffer with 60mM sodium acetate at pH 8.0, before exposure to primary antibodies as described above. Negative controls were also performed excluding primary antibody. Sections were washed thoroughly before the application of secondary antibodies: Alexa Fluor® 488-conjugated goat anti-mouse IgG/IgA/IgM (Invitrogen; at 1:1000 dilution); and for the dual stain, Alexa Fluor® 594-conjugated goat anti-rabbit IgG (Abcam, at 1:1000 dilution), for 30 min at room temperature. After further washes coverslips were applied with Fluoroshield mounting medium with DAPI (Abcam) and sections imaged using an Olympus BX61 fluorescence microscope. Three corneas were analysed for immunolocalization using 3B3, 6C3/K19, and 6C3/ABCB5 antibodies, with five corneas used for immuolocalization using 6C3 and 7D4 antibodies.

**SEAM Differentiation and Immunohistochemistry**

Human iPS cell-derived SEAMs were generated on laminin-511 E8 coated culture plates as described previously (Hayashi et al., 2016; 2017). A time lapse movie of SEAM formation over 21 days is shown as a **Supplementary Movie**. For the current study, SEAMs were first fixed in 4% (wt/vol) paraformaldehyde for 20 min at room temperature after 4 weeks and 6 weeks of differentiation culture. They were then washed 3 times with Tris-buffered saline (TBS). SEAMs were blocked in TBS containing 5% (vol/vol) normal donkey serum and 0.3% (wt/vol) Triton X-100 for 1 hr at room temperature. They were then incubated overnight at 4 °C with 7D4 antibody (Sorrell et al., 1990, 1:20) diluted in TBS containing 1% (vol/vol) normal donkey serum and 0.3% (wt/vol) Triton X-100. SEAMs were then washed three times with TBS for 5 min and subsequently incubated with the secondary antibody: 1:1000 dilution of Alexa Fluor® 488-conjugated goat anti-mouse IgG/IgA/IgM secondary antibody (Invitrogen), for 1 hour at room temperature in the dark. SEAMs were washed again, incubated with Hoechst 33342 for 10 min at room temperature under protection from light, and after final TBS washes, imaged by fluorescence microscopy. Three SEAM cultures were studied at weeks 4, 5 and 6 with multiple SEAMs examined within each sample.


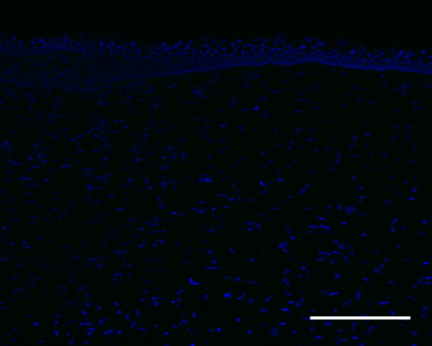

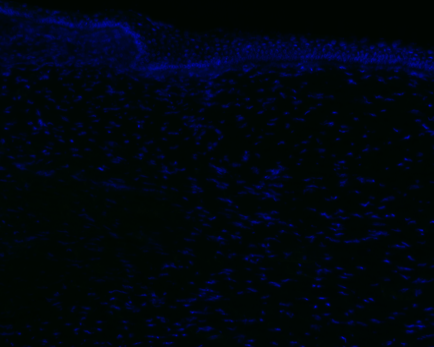

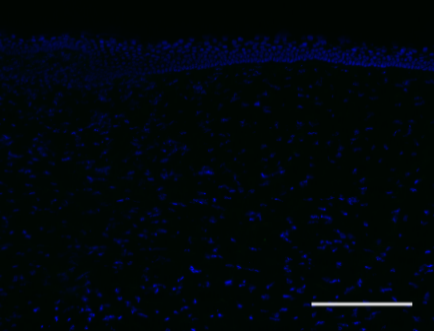

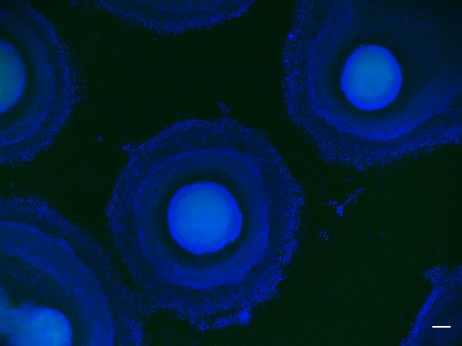


**A**

**B**

**C**

**Supplementary Figure.** Control images for Figures 1 and 2, after omission of primary antibody. (A) Corneal limbus (B) Corneal limbus pre-treated with chondroitinase ABC. (C) A 4 week SEAM. Scale bar represents 200 µm in A and B, and 100 µm in C.
